# Renal Blood Flow Response to Angiotensin 1-7 versus Hypertonic Sodium Chloride 7.5% Administration after Acute Hemorrhagic Shock in Rats

**DOI:** 10.1155/2016/6562017

**Published:** 2016-03-17

**Authors:** Maryam Maleki, Mehdi Nematbakhsh

**Affiliations:** ^1^Water and Electrolytes Research Center, Isfahan University of Medical Sciences, Isfahan 81745, Iran; ^2^Department of Physiology, Isfahan University of Medical Sciences, Isfahan 81745, Iran; ^3^Isfahan MN Institute of Basic and Applied Sciences Research, Isfahan 81745, Iran

## Abstract

*Background.* Angiotensin 1-7 (Ang1-7) plays an important role in renal circulation. Hemorrhagic shock (HS) may cause kidney circulation disturbance, and this study was designed to investigate the renal blood flow (RBF) response to Ang1-7 after HS.* Methods*. 27 male Wistar rats were subjected to blood withdrawal to reduce mean arterial pressure (MAP) to 45 mmHg for 45 min. The animals were treated with saline (group 1), Ang1-7 (300 ng·kg^−1^ min^−1^), Ang1-7 in hypertonic sodium chloride 7.5% (group 3), and hypertonic solution alone (group 4).* Results*. MAP was increased in a time-related fashion (*P*_time_ < 0.0001) in all groups; however, there was a tendency for the increase in MAP in response to hypertonic solution (*P* = 0.09). Ang1-7, hypertonic solution, or combination of both increased RBF in groups 2-4, and these were significantly different from saline group (*P* = 0.05); that is, Ang1-7 leads to a significant increase in RBF to 1.35 ± 0.25 mL/min compared with 0.55 ± 0.12 mL/min in saline group (*P* < 0.05).* Conclusion*. Although Ang1-7 administration unlike hypertonic solution could not elevate MAP after HS, it potentially could increase RBF similar to hypertonic solution. This suggested that Ang1-7 recovers RBF after HS when therapeutic opportunities of hypertonic solution are limited.

## 1. Introduction

Hypovolemic shock, a condition in which tissue perfusion is disturbed to sustain aerobic metabolism [1], occurs due to improper low intravascular volume leading to decrease preload, stroke volume, and cardiac output [2, 3]. Hemorrhagic shock (HS) usually happens when trauma is accompanied with intense blood loss, and in such condition the management of the patients is complex and difficult [4–7]. HS causes poor tissue oxygenation and accumulation of oxygen debt that can lead to multiorgan failure [8, 9] and increases the morbidity rate [10]. On the other hand the kidney function directly depends on renal perfusion pressure (RPP) and this organ is particularly sensitive to HS [11]. HS exacerbates renal damage via decreasing oxygen delivery to the kidney induced hypoxia [12–14] which induces acute kidney injury (AKI) [15]. Delayed diagnosis and treatment may increase morbidity and mortality rates [16]. Therefore during HS necessary interventions are needed to prevent the organ deterioration and function [17].

Renin angiotensin system (RAS) has a pivotal role in kidney function, and it adjusts the body fluid and blood pressure [18, 19]. This system shows both roles of vasoconstriction and vasodilation in kidney and systematic vascular bed, which depend on angiotensin converting enzymes 1 and 2 (ACE1 and ACE2) levels [20, 21]. Angiotensin (Ang) I is hydrolyzed to Ang II via ACE, while hydrolysis of Ang II by ACE2 generates Ang1-7 that subsequently acts upon the Mas receptor [22, 23]. Ang1-7 stimulates nitric oxide (NO) production that may or may not depend on the release of bradykinin [24, 25]. It may also act directly via increasing of prostaglandins to exert the vasodilatory and natriuretic actions [26]. Acute infusion of Ang1-7 increases the glomerular filtration rate (GFR) and renal blood flow (RBF), and it is reported that, in preconstricted afferent arterioles of the rabbit kidney, Ang1-7 induces vasodilation depending on NO [27]. Due to limited use of hypertonic solution in HS, we hypothesized that Ang1-7 administration may promote renal hemodynamic parameters after HS. To test this hypothesis, the RBF responses to Ang1-7, sodium chloride hypertonic solution, or combination of both Ang1-7 plus hypertonic solution compared with vehicle infusion were determined after HS in rats.

## 2. Methods and Materials

### 2.1. Animals

This study was approved in advance by the Ethics Committee of the Isfahan University of Medical Sciences. 27 male Wistar rats weighting 230 to 270 g from Water and Electrolyte Research Center Animal House were used. The animals were housed at a room temperature of 24 ± 1°C with a 12-hour light/dark cycle and fed with rat chow and water ad libitum and allowed 1 week to acclimatize to these conditions.

### 2.2. Surgical Preparation

The animals were anesthetized with urethane (1.7 g·kg^−1^ i.p.; Merck, Germany), and the trachea was cannulated to facilitate air ventilation. Animals were placed in lateral position on a surgical table with heating lamp to control body temperature between 36.5° and 37.5°C. The left jugular vein was exposed, ligated distally, and cannulated with polyethylene tube to infuse the solutions. The left femoral artery was catheterized and the catheter was driven forward into the abdominal aorta below the renal arteries to measure direct blood pressure. The femoral catheter was attached to a pressure transducer and a bridge amplifier (Scientific Concepts, Vic., Melbourne, Australia) to measure mean arterial pressure (MAP) (in fact, we considered renal perfusion pressure as MAP). In order to induce HS, carotid artery was catheterized for blood withdrawal. The bladder also was catheterized to collect urine output. The left kidney was exposed and placed in a cup secured to the operating table. The left renal artery was surrounded by a transit-time ultrasound flow probe (Type 2SB; Transonic Systems, Ithaca, NY, USA) interfaced with a compatible flowmeter (T108; Transonic Systems) to measure direct RBF. Throughout the experiment MAP and RBF were measured continuously and data were recorded as two-second averages via a data acquisition system.

### 2.3. Experimental Protocol

After 30-minute stabilization period, rats underwent controlled HS at MAP 45 mmHg for a period of 45 minutes. Blood withdrawal was carried out in two phases: blood was withdrawn first in 10 min to stabilize MAP at 45 mmHg and second when MAP > 45. The total blood volume withdrawn was measured, and the animals were randomly assigned into the following treatment groups. Group 1 (*n* = 7) as control was subjected to treatment with vehicle (saline). Groups 2 (*n* = 6), 3 (*n* = 9), and 4 (*n* = 5) as treated groups received Ang1-7 (300 ng·kg^−1^ in saline), Ang1-7 in hypertonic sodium chloride 7.5% (5 mL/kg), and hypertonic solution alone. The dose of Ang1-7 (300 ng/kg) was selected based on previous studies to have at least 10 percent change in renal blood flow [28, 29]. The prepared volume of the each infused fluid was equal to the volume of blood withdrawal during HS. The infused fluid was administered as a continuous infusion during the 15 min. Animals were monitored for another 15 min after infusion, and MAP and RBF were measured continuously as described above. Renal vascular resistance was calculated by RPP/RBF ratio.

### 2.4. Statistical Analysis

Data are expressed as mean ± SEM. ANOVA test was applied to analyze the urine weight and serum nitrite level. Repeated measures ANOVA was used to compare the effect of each treatment between groups. *P* value < 0.05 was considered statistically significant.

## 3. Results

### 3.1. Baseline Measurements

The data for MAP, RVR, and RBF were corrected for kidney weight before induction of the HS as basal measurement is tabulated in Table 1. The statistical analyses indicated no significant differences between the groups in basal measurement.

### 3.2. Blood Volume Withdrawal during HS

The animal weights were recorded as 223.0 ± 8.4, 243.2 ± 11.9, 232.5 ± 3.6, and 228.4 ± 8.2 g, and the volumes of blood withdrawal to induce HS were 3.0 ± 0.25, 3.3 ± 0.20, 3.5 ± 0.19, and 3.4 ± 0.26 mL in groups 1 to 4, respectively. There is no statistical difference between the groups in weight (*P* = 0.36) and blood volume withdrawal (*P* = 0.37).

### 3.3. RBF Response to Ang1-7, Sodium Chloride Hypertonic Solution, or Vehicle

**Figure 1:**
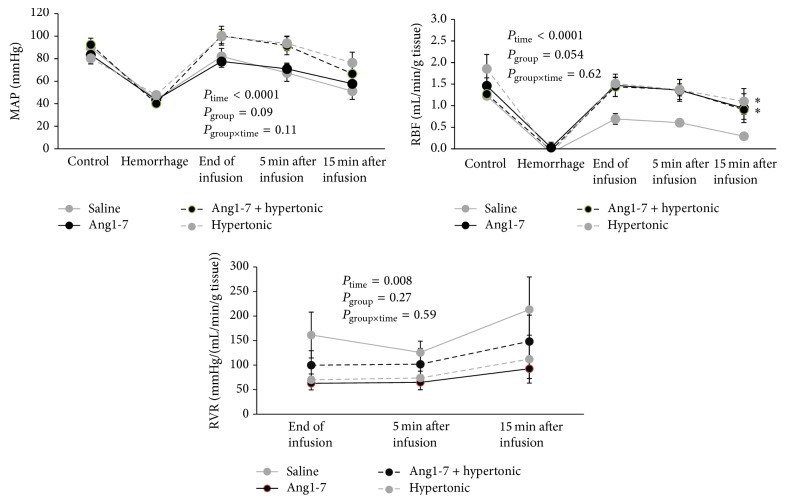
MAP, RBF, and RVR responses to saline, Ang1-7, Ang1-7 + hypertonic saline, and hypertonic saline alone administration after 45 min hemorrhagic shock. Data are shown as mean ± SEM. MAP, mean arterial pressure; RBF, renal blood flow; RVR, renal vascular resistance. *∗* means significant difference between vehicle and the other groups. *P* values were derived from repeated measures for ANOVA.

As blood volume was withdrawn, MAP reduced to about 45 mmHg to induce HS. This pressure was controlled at constant level during 45 min of shock by blood withdrawal. MAP and RBF responses to vehicle, Ang1-7, Ang1-7 plus hypertonic solution, and hypertonic solution alone infusion are shown in Figure 1. MAP and RBF were increased by all the treatment solutions significantly (*P*_time_ < 0.0001). As expected, hypertonic solution provided a better increment for MAP and RBF. However no significant differences were detected in MAP response between the groups. RBF response to Ang1-7, Ang1-7 plus hypertonic solution, and hypertonic solution alone were statistically different from that in vehicle treated group (*P* = 0.05). For example at 5 min after infusion, the RBF response to Ang1-7, Ang1-7 plus hypertonic solution, and hypertonic solution alone were 1.36 ± 0.25, 1.37 ± 0.23, and 1.37 ± 0.14 mL/min/g tissue while this response to vehicle administration was 0.56 ± 0.12 mL/min/g tissue (*P* < 0.05). RVR response during 30 min of shock could not be determined due to the lowest RBF. However, postinfusion records indicated that RVR response in vehicle treated group was greater than other groups insignificantly (*P* = 0.27).

### 3.4. Serum Nitrite Level and Urine Weight

**Figure 2:**
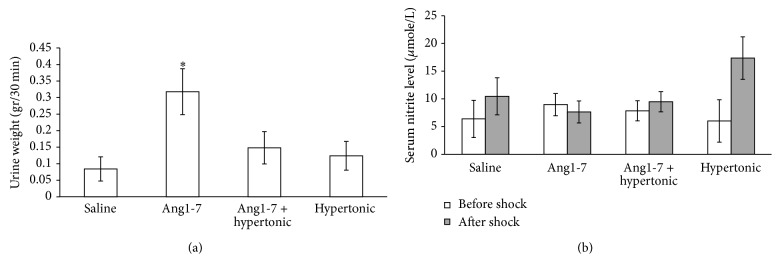
(a) Urine weight response to saline, Ang1-7, Ang1-7 + hypertonic saline, and hypertonic saline alone administration. (b) Serum nitrite level before hemorrhagic shock and 30 minutes after administration of saline, Ang1-7, Ang1-7 + hypertonic saline, and hypertonic saline alone from the end of hemorrhagic shock. Data are shown as mean ± SEM. Ang1-7, Angiotensin 1-7; *∗*, means significant difference between Ang1-7 and saline. *P* value was derived from ANOVA.

During post shock, from starting of infusion until end of experiment the urine was collected and weighted. The result indicated that the urine responses to Ang1-7 infusion were statistically greater than saline group (*P* < 0.05) (Figure 2). To consider nitrite level, no significant differences were observed between the groups neither before nor after the treatments.

## 4. Discussion

Low cardiac output and MAP are considered as predictors of poor outcome in patients which reduces RBF and disturbs kidney functions [30, 31]. Restoration of the hemodynamic status is pivotal for RBF recovery after HS [32]. In this study, RBF responses to different solutions administration in a HS model were determined. RBF responses to Ang1-7, Ang1-7 plus hypertonic solution and hypertonic solution alone were statistically different from that in vehicle treated group while there was a tendency for increasing MAP in response to hypertonic solution groups more than Ang1-7 and vehicle treated groups. In addition, coadministration of Ang1-7 and hypertonic solution did not result in a synergistic effect on RBF compared to administration of Ang1-7 or hypertonic saline alone.

It is expected that both saline and hypertonic solutions produce plasma expansion, blood pressure, and GFR elevation [33]. The suitable MAP level is pivotal to preserve renal function, and at a specific level of MAP, RBF decreases and causes AKI [34]. However, the exact minimum level of MAP to prevent the kidney disturbance still remains unknown [34]. It is reported that MAP above 65 mmHg might be necessary to prevent AKI [35]. Patients with low MAP who also received the highest doses of vasopressors are significantly linked to AKI occurrence [34]. The vasodepressor effect of Ang1-7 mediated by prostaglandins and NO [36–38], and Ang1-7 and its signaling pathways may antagonize the actions of AngII type 1 receptor [39, 40]. One point is important here; the lower concentrations of Ang1-7 are required to stimulate NO compared to that for Ang II acting via the Ang II type 2 vasodilatory receptor [41–43]. Acute administration of Ang1-7 enhances GFR and RBF that reflects the vasorelaxant properties [27]. Ang1-7 also shows the ability to stimulate the formation of NO [44, 45], and it stimulates the phosphorylation of eNOS and the associated kinase Akt in endothelial cells [24]. In addition and similar to bradykinin, Ang1-7 shows a pathway that releases NO [46], and the vasorelaxant actions of Ang1-7 may be reflected by the release of vasoactive prostaglandins, prostacyclin, and PGE2 [47]. It was also reported that the systemically hypotensive effectiveness of Ang1-7 administration was greater in spontaneously hypertensive and renovascular hypertensive than normotensive animal models [37, 48, 49]. Ang1-7 also involves vasopressin release. It is reported that Ang1-7 receptor antagonist (A779) decreased NO concentration to restore the risen vasopressin levels during hemorrhagic shock [50], and it also interacts with the vasopressin V2 receptor [51]. In addition Ang-(1-7) as a potent antidiuretic peptide [52, 53] also influences water excretion [54] possibly by effect on vasopressin system.

Our data from serum nitrite level did not support NO formation by Ang1-7 administration; however it still seems that Ang1-7 increased RBF via formation of NO [44, 45] possibly within the kidney. Our result also did not show the synergistic effect from Ang1-7 plus hypertonic solution because hypertonic solution is liable for large transcapillary absorptive forces which exert maximum intravascular volume expansion immediately at the end of infusion [55] to increase MAP, organ flow, and urine output [56]. MAP elevation by hypertonic solution may increase vascular resistance as Nakamoto et al. reported that hypertension is accompanied with increase of vascular resistance by NO-dependent mechanism created through vascular endothelium [37], and this phenomenon may limit the production of NO by Ang1-7.

## 5. Conclusion

Ang1-7 potentially could increase RBF and urine output due to RVR decreasing after HS. However increased MAP by isotonic normal saline containing Ang1-7 after HS is not similar to sodium chloride hypertonic solution.

## Figures and Tables

**Table 1:** Baseline hemodynamic parameters in 4 experimental groups.

Group	Factor		
	MAP (mmHg)	RBF (mL/min/g tissue)	RVR (mmHg/(mL/min/g tissue))
Saline	89.65 ± 5.96	1.23 ± 0.05	74.04 ± 6.07
Ang1-7	83.42 ± 4.71	1.46 ± 0.18	65.72 ± 15.14
Ang1-7 + hypertonic	92.48 ± 5.69	1.27 ± 0.12	78.04 ± 8.62
Hypertonic	80.38 ± 5.07	1.59 ± 0.33	49.30 ± 9.00
*P*	0.51	0.36	0.25

Data are presented as mean ± SEM. MAP; mean arterial pressure, RVR; renal vascular resistance, RBF; renal blood flow *per *gram kidney weight, Ang1-7; angiotensin 1-7. There were no significant differences between the groups. *P* values were derived from ANOVA.
